# Uncoupling of the center-to-periphery arterial stiffness gradient and pulse pressure amplification in viral pneumonia infection

**DOI:** 10.1186/s12879-023-08650-w

**Published:** 2023-10-05

**Authors:** Lin Jin, Lingheng Wu, Jianxiong Chen, Mengjiao Zhang, Jiali Sun, Cuiqin Shen, Lianfang Du, Xiaoyin She, Zhaojun Li

**Affiliations:** 1 Department of Ultrasound, Guanghua Hospital Affiliated to Shanghai University of Traditional Chinese Medicine, Shanghai, 200052 China; 2 Department of Ultrasound, Jiading Branch of Shanghai General Hospital, Shanghai Jiaotong University School of Medicine, Shanghai, 201812 China; 3 Department of Ultrasound, Shanghai General Hospital of Nanjing Medical University, Shanghai, 200080 China; 4 Department of Ultrasound, Shanghai General Hospital, Shanghai Jiaotong University School of Medicine, Shanghai, 200080 China; 5 Department of Emergency and Critical Care, Jiading Branch of Shanghai General Hospital, Shanghai Jiaotong University School of Medicine, Shanghai, 201812 China

**Keywords:** Aortic stiffness, Arterial stiffness gradient, Blood pressure, Hemodynamics

## Abstract

**Objectives:**

Arterial stiffness is a common manifestation of viral pneumonia infections, including COVID-19. Nevertheless, the relationship between the center-to-periphery arterial stiffness gradient and pulse pressure amplification (PPA) in infectious diseases remains unclear. This study aimed to investigate this relationship utilizing arterial pressure volume index (API) and arterial velocity pulse index (AVI) ratio.

**Methods:**

API/AVI and PPA were measured in 219 participants with COVID-19 and 374 normal participants. Multiple linear regression was used to assess the association of API/AVI and PPA, and restricted cubic spline was used to investigate the non-linear relationship between API/AVI and PPA. Receiver operating characteristic curve (ROC) analysis was used to evaluate the effects of API/AVI in identifying COVID-19 infection and severe stage.

**Results:**

There was a significant J-shaped relationship between API/AVI and PPA in COVID-19 group, while a M-shaped relationship was observed in normal group. API/AVI decreased rapidly as PPA decreased until API/AVI decreased slowly at PPA of 1.07, and then API/AVI decreased slowly again at PPA of 0.78. ROC results showed that API/AVI demonstrated excellent accuracy in identifying COVID-19 infection (AUC = 0.781) and a high specificity (84.88%) in identifying severe stage.

**Conclusions:**

There was a J-shaped association between the API/AVI and PPA in viral infected patients, while a M-shaped relationship in the normal participants. API/AVI is better for identifying infected and uninfected patients, with a high specificity in identifying those in severe stages of the disease. The attenuation or reversal of API/AVI may be associated with the loss of PPA coupling.

**Supplementary Information:**

The online version contains supplementary material available at 10.1186/s12879-023-08650-w.

## Introduction

On May 5th, 2023, the World Health Organization announced that the COVID-19 pandemic no longer constitutes a “public health emergency of international concern”. However, viral infection, represented by COVID-19 can induce a systemic inflammation, leading to cardiovascular complications [1, 2], it is also known to affect artery stiffness and contribute to early vascular aging through various COVID-19-related vascular dysfunction mechanisms [3–5]. Previous studies have demonstrated that the ratio of arterial stiffness between the central elastic aorta and peripheral muscular artery is a stronger predictor of cardiovascular risk than arterial stiffness [6, 7]. Additionally, the central-to peripheral artery stiffness gradient is a key determinant of macrovascular–microvascular interactions [8].

Arterial velocity pulse index (AVI) and arterial pressure volume index (API) are non-invasive indices that provide a reliable measure of arterial stiffness and are strong predictors of cardiovascular risk [9]. AVI is primarily a reflection of central arterial stiffness [10], whereas API is mainly indicative of peripheral muscular artery stiffness [11]. Furthermore, Additionally, the AVI/API ratio can serve as a valuable indicator of the central-to-peripheral arterial stiffness gradient. This measure has the potential to detect central and peripheral vascular dysfunction and thus aid in assessing the relationship between COVID-19 infection and arterial stiffness gradient. Moreover, it may help identify patients who are at risk of a poor disease course.

Aortic-brachial pulse pressure amplification (PPA) is another indirect measure of arterial elasticity that tends to decrease with aging. Pulse pressure (PP) is a composite hemodynamic measure that is strongly predictive of cardiovascular events and is influenced by various factors [12]. Total arterial compliance (TAC), which is affected by arterial size, geometry, and wall stiffness, is one of the primary determinants of PP [13]. However, PPA reflects the discrepancy between central and peripheral blood pressure (BP) (normally, PP is higher in peripheral than central arteries for a similar mean arterial BP) [14]. It is a biomarker of cardiovascular risk stratification that predicts cardiovascular mortality beyond peripheral and central blood pressure measurements [15]. The central-to-peripheral amplification of PP is partly attributable to the stiffness gradient [16]. However, the impact of viral infections, on the central-to-peripheral artery stiffness gradient and PP remains unknown, and the relationship between API/AVI and PPA in patients with COVID-19 infection has not been investigated to date.

Given the evidence that COVID-19 infection is associated with increased arterial stiffness [17], and that increased arterial stiffness typically results in enhanced PPA, we hypothesized that the relationship between API/AVI and PPA would be uncoupled in viral pneumonia infections. Therefore, the aim of this study was to investigate the relationship between arterial stiffness gradients and PPA in patients with COVID-19 infection.

## Methods

### Study population

The study is a cross-sectional study conducted at Shanghai General Hospital Jiading Branch in Shanghai, China, between December 2022 and January 2023, involving participants with normal health status and laboratory-confirmed COVID-19 infection. The study included participants who met the inclusion criteria of having a confirmed COVID-19 infection through a reverse transcription polymerase chain reaction (RT-PCR) swab test. Participants aged below 18 years, those with end-stage renal failure, active malignancy, and previous or current autoimmune diseases were excluded from the study. The baseline COVID-19 severity was determined based on the diagnosis and treatment protocol for COVID-19 (trial tenth edition) released by the National Health Commission of the People’s Republic of China. Mild cases were defined as confirmed cases with mild clinical symptoms and no sign of pneumonia on chest imaging, while moderate cases were defined as confirmed cases with fever, respiratory symptoms, and radiographic evidence of pneumonia. Severe cases were defined as moderate cases with dyspnea or respiratory failure [18, 19]. The control group consisted of individuals who had undergone artery stiffness exams using the same protocol in our hospital between June 2022 and October 2022 and had no history of systemic inflammation or other comorbidities. They were selected based on their similar SBP distributions to the COVID-19 group. After applying the exclusion criteria, a total of 219 participants were included in the final analysis.

Written consents were obtained from all participants. This study was approved by the Ethics Committee of Shanghai General Hospital. All procedures performed in the studies were done in accordance with the ethical standards of the institutional research committee and with the Declaration of Helsinki.

### Physical examination and biochemical tests

Information regarding medical history and current medication was obtained through a questionnaire. In addition, we measured the weight and height of all participants to calculate their body mass index (BMI) using the formula BMI (kg/m^2^) = weight (kg) / height (m)^2^. Venous blood samples were taken at the time of the arterial stiffness measurement to measure white blood cells (WBC), neutrophil, lymphocyte, and platelets (PLT). We also calculated the neutrophil/lymphocyte ratio (NLR) as the ratio between the neutrophil and lymphocyte count.

### Measurement of artery stiffness

At the time of the arterial stiffness measurement, vital signs including blood pressure (BP) and heart rate (HR) were acquired. Participants were asked to rest in a sitting position for 15–30 min before measuring peripheral BP, central BP (central systolic blood pressure (CSBP), and central artery pulse pressure (CAPP)), and artery stiffness values (AVI, API) using the PASESA AVE-2000Pro cuff-oscillometric device (DAIWA Healthcare, Shenzhen, China), as described in previous studies [20, 21]. The average values of three measurements were used for analysis. Pulse pressure (PP) was calculated as systolic blood pressure (SBP) minus diastolic blood pressure (DBP), and center-to-periphery pulse pressure amplification (PPA) was calculated as the ratio of peripheral PP to CAPP. The ratio of API to AVI was used to determine the center-to-periphery (i.e. elastic-to-muscular) artery stiffness gradients, as AVI reflects aortic (elastic) stiffness, while API reflects peripheral (muscular) stiffness [8, 22].

### Statistical analysis

For continuous variables, normality was assessed by histograms, and normally distributed variables are presented as mean ± standard deviation (SD). Nonnormally distributed variables are presented as medians with interquartile ranges. Categorical variables are presented as total numbers and proportions.

Differences in parameters between four groups were compared by the one-way analysis of variance (ANOVA) and post-hoc LSD test for normally continuous variables or Wilcoxon test for nonnormally continuous variables, and the χ^2^ test for categorical variables.

Because of skewed distributions, the data on WBC and PLT were log transformed to acquire normality. Pearson’s analyses of bivariate correlation were applied to measure the strength of associations between API/AVI and other clinical variables.

In addition, to investigate the association between arterial stiffness gradient and hemodynamics, inflammatory factors in COVID-19 infection, stepwise linear regression analyses were performed. Multivariable adjustments were made for the following variables: age, DBP, PP, CAPP, PPA, WBC, NLR.

To investigate the independent relationships between the arterial stiffness gradient, hemodynamics, inflammatory factors and COVID-19 infection, binomial logistic regression analyses were constructed. The following variables were included: age, AVI/API, DBP, CAPP, PP, PPA, NLR.

Because of non-linearity relationships between API/AVI and PPA, restricted cubic spline curves (RCS) were created, and the number of knots was selected on the basis of the model that provided the lowest Akaike information criterion.

Receiver operating characteristic (ROC) analysis was also carried out to examine the API/AVI, PPA levels’ capacity to detect COVID-19, and the cut-off value, sensitivity, and specificity of each marker were determined respectively.

A *p* value less than 0.05 was considered as statistically significant. All analyses were performed using SPSS version 28.0 (IBM Corp, Armonk, NY, USA) and GraphPad Prism version 8.0.0 (GraphPad Software, San Diego, CA, USA) software.

## Results

### Patient characteristics

Baseline clinical, biochemical, artery stiffness and hemodynamics characteristics are presented in Table 1. Briefly, the mean age was 53 ± 18 years and 47% were men. In terms of COVID-19 infection characteristics, 59% were mild, 20% were moderate, and 21% were severe. According to the age division of the World Health Organization, the age of subjects is divided into: young (18 ~ 44 years), middle-aged (45 ~ 59 years), older (≥ 60 years). 53.4% were older in total COVID-19 infection, of which 97.7% were older in moderate and 100% were older in severe COVID-19 infection. While, 137(36.6%) were young, 138(36.9%) were middle-aged, and 99(26.5%) were older in control group.

**Table 1:** Baseline characteristics of the study participants

Variables	Control (n = 374)	Mild (n = 129)	Moderate (n = 43)	Severe (n = 47)	*p* value
**Demographics**					
Age, years	49.37 ± 13.88	45.01 ± 15.75^a^	76.88 ± 9.78 ^ab^	83.96 ± 8.12 ^abc^	<0.001
Male, n (%)	176 (47.1%)	45(34.9%)	25(58.1%)	30(63.8%)	0.002
Hypertension, n (%)	172(58.7%)	50(17.1%)	36(12.3%)	35(11.9%)	<0.001
Diabetes, n (%)	32(8.6%)	126 (97.7%)	35 (81.4%)	41 (87.2%)	<0.001
**Signs**					
BMI, kg/m^2^	23.45 ± 3.29	23.55 ± 3.49	23.33 ± 4.22	22.81 ± 3.82	0.634
HR, bpm	78.46 ± 12.15	78.57 ± 12.32	80.33 ± 11.89	88.11 ± 16.95 ^ab^	<0.001
SBP, mm Hg	129.92 ± 16.62	125.12 ± 16.72	128.26 ± 21.83	128.00 ± 19.81	0.061
DBP, mm Hg	83.26 ± 12.42	80.34 ± 11.95 ^a^	75.79 ± 13.73 ^ab^	73.96 ± 14.90 ^ab^	<0.001
PP, mmHg	46.66 ± 14.05	44.78 ± 12.27	52.47 ± 17.67	54.04 ± 17.27 ^ab^	<0.001
CSBP, mm Hg	127.77 ± 15.54	117.60 ± 15.19 ^a^	124.37 ± 17.77^b^	123.36 ± 15.74^b^	<0.001
CAPP, mm Hg	39.60 ± 10.30	37.12 ± 9.71 ^a^	51.19 ± 12.75 ^ab^	51.15 ± 11.45 ^ab^	<0.001
PPA	1.17 ± 0.17	1.20 ± 0.09 ^a^	1.00 ± 0.15 ^ab^	1.03 ± 0.15 ^abc^	<0.001
**Biochemical analysis**					
WBC, /L	0.82 ± 0.13	0.82 ± 0.12	0.82 ± 0.19	0.95 ± 0.19^abc^	<0.001
Neutrophil (%)	60.19 ± 11.19	61.06 ± 10.39	68.09 ± 12.57^ab^	79.57 ± 10.10 ^abc^	<0.001
Lymphocyte (%)	29.90(23.10,36.83)	29.20(24.05,34.90)	20.80(14.20,26.10)^ab^	10.20(6.20,15.10)^abc^	<0.001
NLR	1.98(1.42,2.89)	2.08(1.51,2.79)	3.32(2.30,5.60)^ab^	7.88(4.98,14.43)^abc^	<0.001
Hemoglobin, g/dL	136.44 ± 17.49	135.10 ± 17.67	108.28 ± 21.34^ab^	108.98 ± 18.01^ab^	<0.001
PLT, /L	2.34 ± 0.12	2.36 ± 0.13	2.27 ± 0.21	2.21 ± 0.21^ab^	<0.001
**Arterial stiffness indices**					
AVI	12.97 ± 5.28	15.12 ± 5.18^a^	22.16 ± 5.36 ^ab^	21.06 ± 6.45 ^ab^	<0.001
API	29.00 ± 6.81	24.60 ± 4.92 ^a^	27.98 ± 6.39 ^b^	28.91 ± 6.16 ^b^	<0.001
API/AVI	2.28(1.72,3.20)	1.67(1.39,2.00) ^a^	1.33(0.94,1.52) ^ab^	1.38(1.04,1.75) ^a^	<0.001

Data are presented as means ± SD or medians (interquartile range) for continuous variables or number (%) for categorized variables. BMI, body mass index; SBP, systolic blood pressure; DBP, diastolic blood pressure; HR, heart rate; WBC, white blood cells; PLT, platelets; AVI, Arterial velocity pulse index; API, arterial pressure volume index; NLR, neutrophil/lymphocyte ratio; PPA, pulse pressure amplification; PP, pulse pressure

Because of skewness, several variables were transformed to acquire normality. WBC and PLT were log transformed. Compared with the control group, ^a^p < 0.05; Compared with the mild group, ^b^p < 0.05; Compared with the Moderate group, ^c^p < 0.05

Table 1 also outlined artery stiffness and hemodynamics characteristics, as API/AVI, and PPA. In mild COVID-19 infection patients had lower API/AVI and higher PPA, while in moderate and severe COVID-19 infection patients had lower API/AVI and lower PPA compared to the normal group. Because age was an important determinant of arterial stiffness, Table 2 outlined PPA and API/AVI characteristics in older groups.

**Table 2:** Comparison of arterial stiffness parameters in older group

	Controls (n = 99)	Mild (n = 28)	Moderate (n = 42)	Severe (n = 47)	*p* value
API/AVI	1.83(1.61,2.29)	1.56(1.29,1.76)^a^	1.33(0.94,1.54) ^a^	1.38(1.04,1.75)^a^	< 0.001
PPA	1.17 ± 0.16	1.16 ± 0.06	1.00 ± 0.14^ab^	1.03 ± 0.15^ab^	< 0.001

Compared with the control group, ^a^p < 0.05; Compared with the mild group, ^b^p < 0.05; Compared with the Moderate group, ^c^p < 0.05

### Relationships between hemodynamics, clinical parameters and API/AVI in patients with COVID-19

**Fig. 1:**
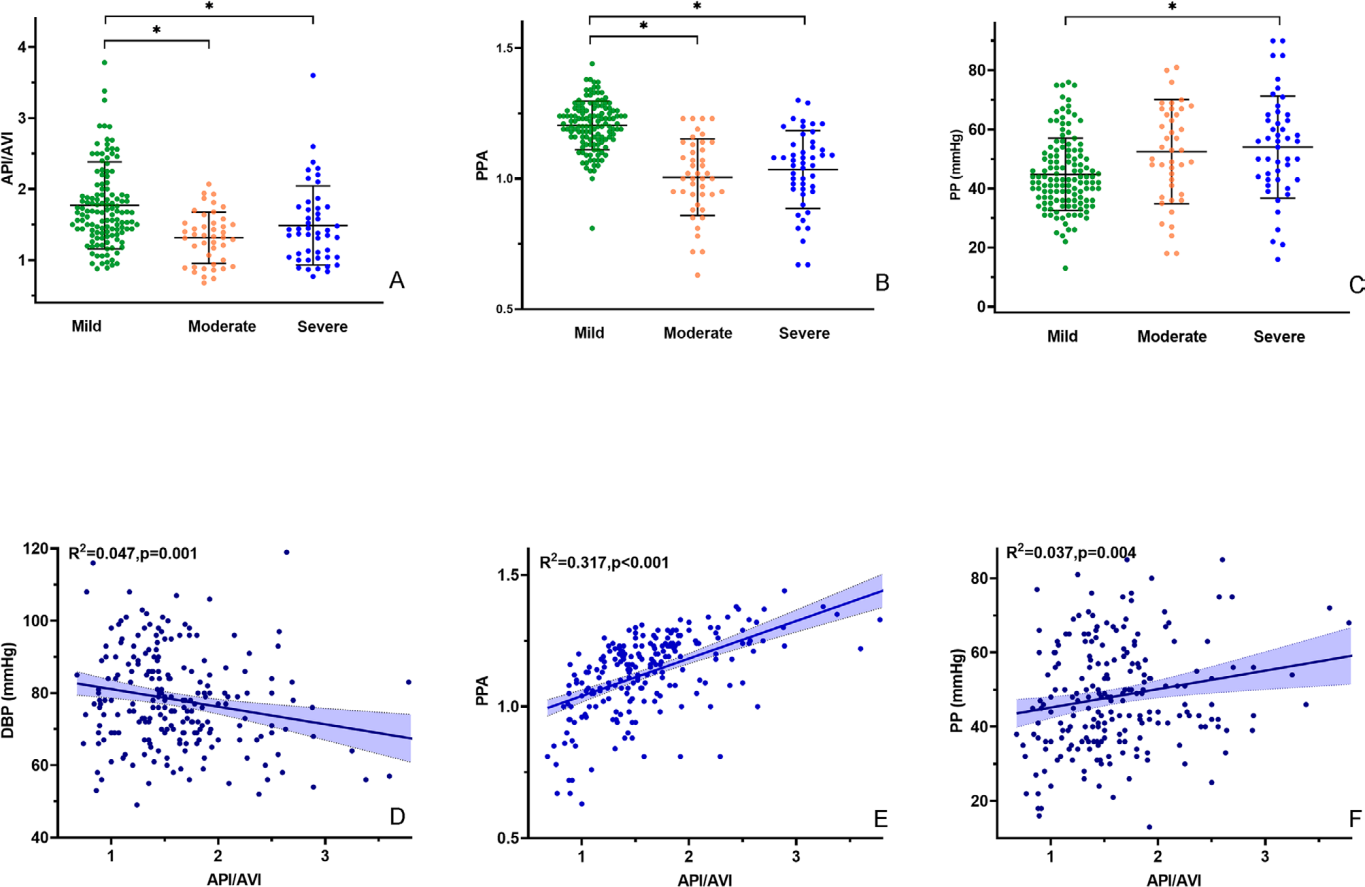
Distribution of AVI/API, PPA and PP and association between API/AVI and hemodynamics indices in COVID-19. Violin plots depict the mean (black line), SD (black line), of API/AVI **(A)**, PPA **(B)** and PP **(C)**. API/AVI were significantly associated with DBP **(D)**, correlated with PPA **(E)** and PP **(F)** in COVID-19 patients. DBP, diastolic blood pressure; PPA, pulse pressure amplification; PP, pulse pressure

In unadjusted analyses, age, DBP, PP and PPA showed significant associations with API/AVI in COVID-19 patients (Supplementary Table 1) (Fig. 1). But after multivariable adjustment in linear regression analyses, this association changed such that only PPA and DBP was independently associated with API/AVI (Table 3). No as sociation with age, PP and API/AVI was observed after multivariable adjustments.

**Table 3:** Multiple linear regression analysis of API/AVI in COVID-19

Item	β	95%CI	VIF	*p* value
PPA	1.941	1.520, 2.362	1.029	<0.001
DBP	-0.005	-0.010, 0.000	1.029	0.031

β is the regression coefficient, VIF is the valiance inflation factor

The following variables were included in the analysis: age, DBP, PP, CAPP, PPA, WBC, NLR

### Relationships between API/AVI and PPA

**Fig. 2:**
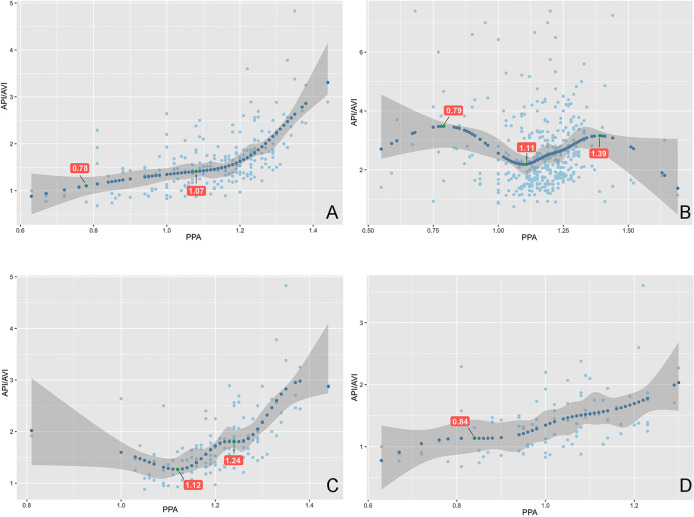
Correlations between API/AVI and PPA in COVID-19 and normal group based on Restricted Cubic Spline analysis. There was a significant J-shaped relationship between API/AVI and PPA in total COVID-19 group **(A)**. API/AVI decreased rapidly as PPA decreased until API/AVI decreased slowly at PPA of 1.07, and then API/AVI decreased slowly again at PPA of 0.78. There was a significant M-shaped relationship between API/AVI and PPA in normal group **(B)**. There was a significant J-shaped relationship between API/AVI and PPA in Mild COVID-19 group **(C)**, and in Moderate and Severe COVID-19 group **(D)**

In order to further explore the relationship between API/AVI and PPA, the RCS curves was drawn. The results showed that: There was a significant J-shaped relationship between API/AVI and PPA in COVID-19 (Fig. 2A). Specifically, we observed that API/AVI decreased rapidly as PPA decreased, until reaching a minimum at PPA of 1.07, after which API/AVI decreased more slowly. Another inflection point was observed at PPA of 0.78, after which API/AVI decreased slowly once again. However, a M-shaped relationship between API/AVI and PPA was observed in the normal patients (Fig. 2B). Furthermore, there was also a significant J-shaped relationship between API/AVI and PPA in Mild COVID-19 group, and in Moderate and Severe COVID-19 group (Fig. 2C and D).

### Association of arterial stiffness gradient with COVID-19

The ordinal logistic regression analysis of the correlation between arterial stiffness gradient and COVID-19 infected severity was presented in Table 4. The model adjusted age, DBP, PP, PPA, API/AVI, CAPP and NLR. The results showed thar a change of age was associated with an increase in the odds of 0.994 in mild COVID-19, and 1.224 in moderate and severe COVID-19 than normal. While, a change of API/AVI was associated with an increase in the odds of 0.160 in mild COVID-19, and 0.104 in moderate and severe COVID-19 than normal.

**Table 4:** **Logistic regression analysis of arterial stiffness gradient associated with COVID-19 Severity**

Variable	Mild		Moderate and Severe	
	OR (95% CI)	*p* value	OR (95% CI)	*p* value
age	0.944(0.920,0.968)	<0.001	1.224(1.129,1.327)	<0.001
API/AVI	0.160(0.092,0.278)	<0.001	0.104(0.029,0.376)	0.001
DBP	0.977(0.953,1.002)	0.071	0.952(0.910,0.996)	0.032
NLR	0.961(0.850,1.086)	0.526	1.149(1.037,1.274)	0.008

CI is the confidence interval, OR is the odds ratio

To control for age confounders, logistic regression analyses were performed in the older age group. API/AVI still had a significant association with the COVID-19 infected in older [OR (95% CI), 0.203(0.081, 0.514)] (Table 5).

**Table 5:** Logistic regression analysis of arterial stiffness gradient associated with COVID-19 in older

Variable	β	OR(95% CI)	*p* value
age	0.235	1.264(1.173, 1.363)	<0.001
API/AVI	-1.593	0.203(0.081, 0.514)	0.001
PPA	-2.807	0.060(0.003, 1.068)	0.056

### ROC curve of API/AVI, PPA in identification of COVID-19 infected status

**Fig. 3:**
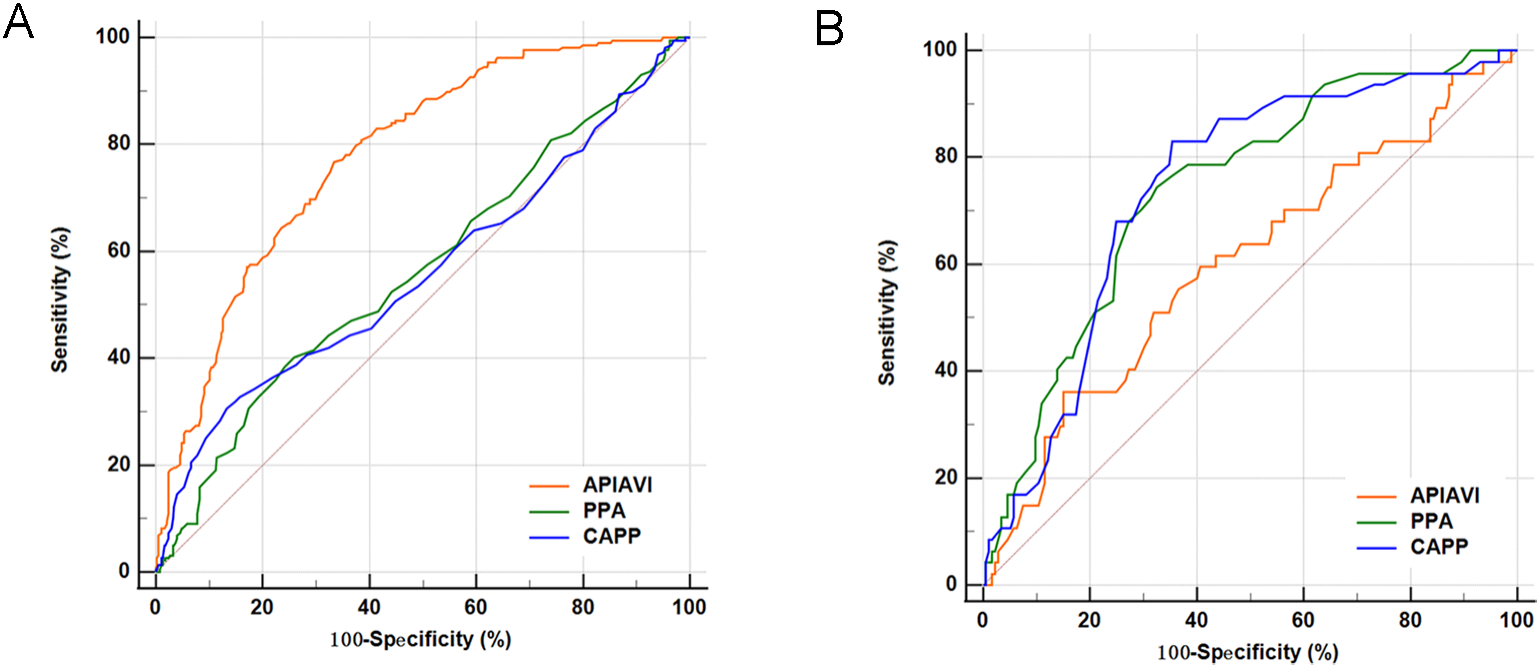
Receiver operating characteristics (ROC) curve of API/AVI, PPA, and CAPP in identification of COVID-19 **(A)** and severe infected **(B)**

A ROC analysis was conducted to determine the API/AVI and PPA cutoff value that would identify the COVID-19 infected from uninfected patients. As depicted in Fig. 3A, the optimal API/AVI cutoff value to indicate COVID-19 infected was 1.87, with a sensitivity 76.71% and specificity 66.58%, and an area under the curve of 0.781 (95% CI 0.746–0.814). The cut-off of CAPP is 50 mmHg with sensitivity 30.59% and specificity 86.63%. The cut-off of PPA is 1.1 with sensitivity 38.36% and specificity 75.94%.

In addition, ROC curves to predict patients with severe COVID-19 stage were plotted (Fig. 3B). The area under the ROC of API/AVI were 0.600 (95% CI: 0.532–0.666), CAPP were 0.741 (95% CI: 0.677–0.797), PPA were 0.739 (95% CI: 0.676–0.796), respectively. The cut-off of API/AVI is 0.86 with specificity 84.88%. The cut-off of CAPP is 41 mmHg with sensitivity 82.98%. The cut-off of PPA is 1.12 with sensitivity 74.47% and specificity 67.44%.

## Discussion

In the present investigation, the association between API/AVI ratio and PPA in COVID-19 patients was investigated. In comparison to normal group, PPA initially increased in the mild COVID-19, followed by a steady decrease in moderate to severe COVID-19. While, API/AVI initially decreased in the mild COVID-19, followed by a further decrease in moderate to severe COVID-19. Furthermore, COVID-19 patients had a J-shaped association between the API/AVI and PPA, while a M-shaped relationship between API/AVI and PPA was observed in the normal patients.

As previously noted, COVID-19 infection has been associated with vascular dysfunction [23, 24], a finding that was also observed in this study, with COVID-19 patients exhibiting elevated AVI. However, interestingly, the API/AVI ratio initially decreased in mild COVID-19 cases, followed by a further decrease in moderate to severe cases. This may be due to systemic microcirculatory dysfunction in different vascular beds caused by COVID-19-induced endotheliitis [25]. Endothelial dysfunction can shift the vascular equilibrium towards more vasoconstriction, leading to organ ischemia [26]. The decrease in the central-to-peripheral arterial stiffness gradient may facilitate protection of vital organ perfusion.

The observed increase in center-to-periphery arterial stiffness gradient in COVID-19 patients can have detrimental effects on various organs, leading to left ventricular dysfunction [27], amplified pulse pressure, reduced coronary perfusion, and brain microvascular injury, ultimately contributing to a poor prognosis [28]. Surprisingly, this study found that PPA initially increased in mild COVID-19 patients, followed by a steady decrease in moderate to severe cases. Moreover, there was an independent association between PPA and API/AVI, and a J-shaped association between the two. The decrease in API/AVI was rapid with decreasing PPA until PPA reached 1.07, after which it decreased slowly until PPA of 0.78. However, this relationship was not found in the normal group. We hypothesized that the relationship was associated with COVID-19 infection, severity, and PPA uncoupling.

It is possible that COVID-19 may lead to large artery stiffening as it is a multisystem disease with hyperinflammation and altered immune response, which may have detrimental effects on the systemic vasculature in both the short and long term [17, 29]. This may result from changes in arterial wall tissue due to increased inflammatory cells and inflammatory edema. Additionally, the close relationship between arterial stiffness and the severity of chronic inflammation has been demonstrated in several systemic inflammatory diseases [30, 31]. Another possible mechanism is that our study was conducted during the early stages of COVID-19 infection. The stress response, which is a series of changes in the neuro-endocrine system, organs, and internal environment of the organism with COVID-19, may play a role in promoting the organism’s ability to mobilize itself to preserve life and restore the structural and functional integrity of tissues and organs [32–34]. The diminished or inversion of the stiffness gradient in COVID-19 may therefore increase diastolic blood flow to reduce organ ischemia and injury.

It is important to note that in this study, COVID-19 patients had a higher prevalence of diabetes, a condition that is associated with systemic inflammation and may weaken the body’s ability to resist the coronavirus. It has been consistently shown that cardiovascular risk factors such as hypertension, diabetes, and dyslipidemia significantly increase the risk of severe COVID-19, possibly due to the pre-existing structural and functional changes in organs caused by prolonged blood pressure elevation and metabolic alterations, which may compromise their ability to withstand the virus [35].

The following are the study’s limitations. First, since this is a cross-sectional study, it provides no data on longitudinal API/AVI and PPA changes in variables associated with COVID-19 infection. And the mid- and long-term effects of COVID-19 on API/AVI and PPA will be evaluate in future. Second, this study only included patients diagnosed with COVID-19 viral pneumonia. So a selection bias existed and our sample could not fully represent the general viral pneumonia infection population in the real world. Therefore, extrapolation of our results to other viral pneumonia infection populations should be done cautiously. Third, the mechanism of the M-ship relationship between API/AVI and PPA in normal participants is not fully understood. The lack of data on the high PPA range in the normal group may affect the relationship leading to M-shaped. Longer and larger longitudinal studies will further evaluate the non-linear relationship between PPA and API/AVI as a later observation for cardiovascular risk assessment.

## Conclusion

In conclusion, there was a J-shaped association between the API/AVI and PPA in COVID-19 infected patients, and a M-shaped relationship in the normal participants. API/AVI is better for identifying infected and uninfected patients, with a high specificity to identify patients with severe stage. The attenuation/reversal of API/AVI may be associated with the loss of coupling of PPA.

### Electronic supplementary material

Below is the link to the electronic supplementary material.

Supplementary Material 1

## Data Availability

The datasets used and/or analysed during the current study available from the corresponding author on reasonable request.

## References

[1] European Society of Cardiology guidance for the (2022). Diagnosis and management of cardiovascular disease during the COVID-19 pandemic: part 1-epidemiology, pathophysiology, and diagnosis. Eur Heart J.

[2] Olsen FJ, Lassen MCH, Skaarup KG, Christensen J, Davidovski FS, Alhakak AS, Sengeløv M, Nielsen AB, Johansen ND, Graff C (2022). Myocardial work in patients hospitalized with COVID-19: relation to biomarkers, COVID-19 severity, and all-cause mortality. J Am Heart Assoc.

[3] Schnaubelt S, Oppenauer J, Tihanyi D, Mueller M, Maldonado-Gonzalez E, Zejnilovic S, Haslacher H, Perkmann T, Strassl R, Anders S (2021). Arterial stiffness in acute COVID-19 and potential associations with clinical outcome. J Intern Med.

[4] Szeghy RE, Province VM, Stute NL, Augenreich MA, Koontz LK, Stickford JL, Stickford ASL, Ratchford SM (2022). Carotid stiffness, intima-media thickness and aortic augmentation index among adults with SARS-CoV-2. Exp Physiol.

[5] Lambadiari V, Mitrakou A, Kountouri A, Thymis J, Katogiannis K, Korakas E, Varlamos C, Andreadou I, Tsoumani M, Triantafyllidi H (2021). Association of COVID-19 with impaired endothelial glycocalyx, vascular function and myocardial deformation 4 months after infection. Eur J Heart Fail.

[6] Fortier C, Mac-Way F, Desmeules S, Marquis K, De Serres SA, Lebel M, Boutouyrie P, Agharazii M (2015). Aortic-brachial stiffness mismatch and mortality in dialysis population. Hypertension.

[7] Covic A, Siriopol D (2015). Pulse wave velocity ratio: the new gold standard for measuring arterial stiffness. Hypertension.

[8] London GM, Pannier B, Safar ME (2019). Arterial stiffness gradient, systemic reflection coefficient, and pulsatile pressure Wave Transmission in essential hypertension. Hypertension.

[9] Jin L, Tong L, Shen C, Du L, Mao J, Liu L, Li Z. Association of Arterial Stiffness Indices with Framingham Cardiovascular Disease Risk Score. *RCM* 2022, 23(8):287. 10.31083/j.rcm2308287 PMC11266941 39076621

[10] Ueda T, Miura S, Suematsu Y, Shiga Y, Kuwano T, Sugihara M, Ike A, Iwata A, Nishikawa H, Fujimi K (2016). Association of arterial pressure volume Index with the Presence of significantly stenosed coronary vessels. J Clin Med Res.

[11] Okamoto M, Nakamura F, Musha T, Kobayashi Y (2016). Association between novel arterial stiffness indices and risk factors of cardiovascular disease. BMC Cardiovasc Disord.

[12] Nilsson PM, Cederholm J, Eeg-Olofsson K, Eliasson B, Zethelius B, Gudbjörnsdóttir S (2009). Pulse pressure strongly predicts cardiovascular disease risk in patients with type 2 diabetes from the Swedish National Diabetes Register (NDR). Diabetes Metab.

[13] Chirinos JA, Segers P, Gillebert TC, De Buyzere ML, Van Daele CM, Khan ZA, Khawar U, De Bacquer D, Rietzschel ER (2013). Central pulse pressure and its hemodynamic determinants in middle-aged adults with impaired fasting glucose and diabetes: the Asklepios study. Diabetes Care.

[14] Avolio AP, Van Bortel LM, Boutouyrie P, Cockcroft JR, McEniery CM, Protogerou AD, Roman MJ, Safar ME, Segers P, Smulyan H (2009). Role of pulse pressure amplification in arterial hypertension: experts’ opinion and review of the data. Hypertension.

[15] Benetos A, Thomas F, Joly L, Blacher J, Pannier B, Labat C, Salvi P, Smulyan H, Safar ME (2010). Pulse pressure amplification a mechanical biomarker of cardiovascular risk. J Am Coll Cardiol.

[16] Kanazawa M, Fukuyama H, Kinefuchi Y, Takiguchi M, Suzuki T (2003). Relationship between aortic-to-radial arterial pressure gradient after cardiopulmonary bypass and changes in arterial elasticity. Anesthesiology.

[17] Saeed S, Mancia G (2021). Arterial stiffness and COVID-19: a bidirectional cause-effect relationship. J Clin Hypertens (Greenwich).

[18] Commission NH (2023). Diagnosis and treatment protocol for novel coronavirus pneumonia (trial 10th version). Chin J Clin Infect Dis.

[19] Faqin Lv J, Wang X, Yu A, Yang J-B, Liu L, Qian H, Xu L, Cui M, Xie X, Liu (2020). Chinese Expert Consensus on critical care Ultrasound Applications at COVID-19 pandemic. Adv Ultrasound Diagnosis Therapy.

[20] Sasaki-Nakashima R, Kino T, Chen L, Doi H, Minegishi S, Abe K, Sugano T, Taguri M, Ishigami T (2017). Successful prediction of cardiovascular risk by new non-invasive vascular indexes using suprasystolic cuff oscillometric waveform analysis. J Cardiol.

[21] Jin L, Chen J, Zhang M, Sha L, Cao M, Tong L, Chen Q, Shen C, Du L, Li Z et al. Relationship of arterial stiffness and central hemodynamics with cardiovascular risk in hypertension. Am J Hypertens 2023. 10.1093/ajh/hpad005 36645322

[22] Hashimoto J, Ito S (2013). Aortic stiffness determines diastolic blood flow reversal in the descending thoracic aorta: potential implication for retrograde embolic stroke in hypertension. Hypertension.

[23] Nägele MP, Haubner B, Tanner FC, Ruschitzka F, Flammer AJ (2020). Endothelial dysfunction in COVID-19: current findings and therapeutic implications. Atherosclerosis.

[24] Kaur S, Tripathi DM, Yadav A (2020). The Enigma of Endothelium in COVID-19. Front Physiol.

[25] Varga Z, Flammer AJ, Steiger P, Haberecker M, Andermatt R, Zinkernagel AS, Mehra MR, Schuepbach RA, Ruschitzka F, Moch H (2020). Endothelial cell infection and endotheliitis in COVID-19. Lancet.

[26] Bonetti PO, Lerman LO, Lerman A (2003). Endothelial dysfunction: a marker of atherosclerotic risk. Arterioscler Thromb Vasc Biol.

[27] Tomiyama H, Shiina K (2020). State of the art review: brachial-ankle PWV. J Atheroscler Thromb.

[28] Vlachopoulos C, Xaplanteris P, Aboyans V, Brodmann M, Cífková R, Cosentino F, De Carlo M, Gallino A, Landmesser U, Laurent S (2015). The role of vascular biomarkers for primary and secondary prevention. A position paper from the European Society of Cardiology Working Group on peripheral circulation: endorsed by the Association for Research into arterial structure and physiology (ARTERY) society. Atherosclerosis.

[29] Wang B, Zhang L, Zhang D, Yuan H, Wu C, Zhang Y, He L, Wang R, Wang J (2020). Mingxing Xie: Bedside Ultrasound in Assessment of 510 severe and critical patients with COVID-19 pneumonia in Wuhan, China. Adv Ultrasound Diagnosis Therapy.

[30] Zanoli L, Briet M, Empana JP, Cunha PG, Mäki-Petäjä KM, Protogerou AD, Tedgui A, Touyz RM, Schiffrin EL, Spronck B (2020). Vascular consequences of inflammation: a position statement from the ESH Working Group on Vascular structure and function and the ARTERY Society. J Hypertens.

[31] Zanoli L, Boutouyrie P, Fatuzzo P, Granata A, Lentini P, Oztürk K, Cappello M, Theocharidou E, Tuttolomondo A, Pinto A et al. Inflammation and aortic stiffness: an individual Participant Data Meta-Analysis in patients with inflammatory bowel disease. J Am Heart Assoc 2017, 6(10). 10.1161/JAHA.117.007003 PMC5721883 29018026

[32] Wang D, Hu B, Hu C, Zhu F, Liu X, Zhang J, Wang B, Xiang H, Cheng Z, Xiong Y (2020). Clinical characteristics of 138 hospitalized patients with 2019 Novel Coronavirus-Infected pneumonia in Wuhan, China. JAMA.

[33] Russell G, Lightman S (2019). The human stress response. Nat Rev Endocrinol.

[34] Cianfarani S, Pampanini V (2023). The impact of stress on Health in Childhood and Adolescence in the era of the COVID-19 pandemic. Horm Res Paediatr.

[35] Zhou X, Lu H, Sang M, Qiu S, Yuan Y, Wu T, Chen J, Sun Z (2023). Impaired antibody response to inactivated COVID-19 vaccines in hospitalized patients with type 2 diabetes. Hum Vaccin Immunother.

