# Supplementary material for: Uncoupling of the center-to-periphery arterial stiffness gradient and pulse pressure amplification in viral pneumonia infection
Source: BMC Infect Dis. 2023 Oct 5;23:657. doi: 10.1186/s12879-023-08650-w (PMC10552441; doi:10.1186/s12879-023-08650-w)
Supplement: Supplementary file 1 — Supplementary Material 1 [file 12879_2023_8650_MOESM1_ESM.docx]

**Supplementary Table 1. Correlation between API/AVI, hemodynamics and inflammation**

|  | Total | | Control | | COVID-19 | |
| --- | --- | --- | --- | --- | --- | --- |
|  | r | *p* value | r | *p* value | r | *P* value |
| age | -0.374 | <0.001 | -0.347 | <0.001 | -0.352 | <0.001 |
| CSBP | -0.250 | <0.001 | 0.251 | <0.001 | -0.042 | 0.541 |
| CAPP | -0.100 | 0.015 | -0.060 | 0.250 | -0.029 | 0.673 |
| SBP | 0.000 | 0.993 | -0.063 | 0.223 | 0.003 | 0.963 |
| DBP | 0.021 | 0.611 | -0.025 | 0.634 | -0.217 | 0.001 |
| PP | -0.019 | 0.641 | -0.053 | 0.307 | 0.193 | 0.004 |
| WBC | 0.122 | 0.008 | -0.077 | 0.192 | -0.103 | 0.171 |
| NLR | 0.167 | <0.001 | -0.156 | 0.008 | -0.047 | 0.534 |
| PPA | 0.115 | 0.005 | -0.044 | 0.395 | 0.563 | <0.001 |
